# Protein Tyrosine Phosphatases in Neuroblastoma: Emerging Roles as Biomarkers and Therapeutic Targets

**DOI:** 10.3389/fcell.2021.811297

**Published:** 2021-12-08

**Authors:** Caroline E. Nunes-Xavier, Laura Zaldumbide, Lorena Mosteiro, Ricardo López-Almaraz, Nagore García de Andoin, Pablo Aguirre, Maite Emaldi, Leire Torices, José I. López, Rafael Pulido

**Affiliations:** ^1^ Biomarkers in Cancer Unit, Biocruces Bizkaia Health Research Institute, Barakaldo, Spain; ^2^ Department of Tumor Biology, Institute for Cancer Research, Oslo University Hospital Radiumhospitalet, Oslo, Norway; ^3^ Department of Pathology, Cruces University Hospital, Barakaldo, Spain; ^4^ Pediatric Oncology and Hematology, Cruces University Hospital, Barakaldo, Spain; ^5^ Unit of Pediatric Oncohematology, Donostia University Hospital, San Sebastian, Spain; ^6^ Department of Pathology, Donostia University Hospital, San Sebastian, Spain; ^7^ IKERBASQUE, Basque Foundation for Science, Bilbao, Spain

**Keywords:** protein tyrosine phosphatases, neuroblastoma, biomarker, cell signaling, phosphorylation, dephosphorylation

## Abstract

Neuroblastoma is a type of cancer intimately related with early development and differentiation of neuroendocrine cells, and constitutes one of the pediatric cancers with higher incidence and mortality. Protein tyrosine phosphatases (PTPs) are key regulators of cell growth and differentiation by their direct effect on tyrosine dephosphorylation of specific protein substrates, exerting major functions in the modulation of intracellular signaling during neuron development in response to external cues driving cell proliferation, survival, and differentiation. We review here the current knowledge on the role of PTPs in neuroblastoma cell growth, survival, and differentiation. The potential of PTPs as biomarkers and molecular targets for inhibition in neuroblastoma therapies is discussed.

## Introduction

Neuroblastoma is the most common extracranial solid tumor diagnosed in infants and the paediatric cancer with higher risk of death, with high-risk neuroblastoma (about 50% of neuroblastoma cases) showing a survival rate of about 50% (Ward et al., 2014; Siegel et al., 2020). Neuroblastomas display high clinical heterogeneity, from tumors that spontaneously regress to metastatic tumors refractory to multi-therapies (Matthay et al., 2016; Brodeur, 2018; Kholodenko et al., 2018; Tsubota and Kadomatsu, 2018). Neuroblastoma tumors arise from endocrine neural crest precursor cells during aberrant development of sympathetic neuronal cells early in life. This makes neuroblastoma potentially actionable from the perspective of the developmental biology of neuroendocrine cells (Cheung and Dyer, 2013; Fletcher et al., 2018). However, the low number of mutations found in neuroblastoma tumors at diagnosis, their high diversity, and the paediatric nature of the patients, have been handicaps for the identification and validation of actionable molecular targets. In this context, anti-disialoganglioside GD2 monoclonal antibody immunotherapy is the only current targeted therapy for high-risk neuroblastoma (Pastor and Mousa, 2019; Moreno et al., 2020). Protein phosphorylation/dephosphorylation plays an important role in the control of neuroblastoma cell growth and transformation, and inhibition of oncogenic protein kinases is actively being tested in paediatric neuroblastoma clinical trials (Stafman and Beierle, 2016; Applebaum et al., 2017; Berlanga et al., 2017; Pacenta and Macy, 2018; Moreno et al., 2020). Distinct groups of protein phosphatases exist that play relevant roles in human disease (Chen et al., 2017), and their significance as molecular targets in cancer, including paediatric cancers, is gaining attention. For instance, the modulation of the activity of the serine/threonine phosphatase PP2A is being explored as an intervention in neuroblastoma cell survival and tumor growth (Williams et al., 2019).

Protein tyrosine phosphatases (PTPs) dephosphorylate key homeostatic phospho-proteins and are major regulators of developmental processes, including neuronal survival and differentiation (Hendriks et al., 2013; Hendriks and Pulido, 2013; Tonks, 2013; Hale et al., 2017). Accordingly, specific inhibitors of PTP catalytic activity are under scrutiny for their therapeutic application in human cancer (Ríos et al., 2014; Frankson et al., 2017; Lazo et al., 2018). In neuroblastoma cells, general inhibition of PTPs by vanadium derivatives limits cell growth and triggers apoptosis (Clark et al., 2013; Clark et al., 2015). The PTP superfamily includes two major subgroups, the classical PTPs, which specifically dephosphorylate phospho-tyrosine residues from proteins; and the dual-specificity PTPs (DUSPs), which dephosphorylate phospho-serine/threonine/tyrosine residues, in many cases from the activation loop of MAP kinases, as well as non-protein substrates (Alonso et al., 2004; Julien et al., 2011; Nunes-Xavier et al., 2011; Alonso et al., 2016). The involvement of DUSPs in neuroblastoma cell growth and differentiation has been recently reviewed (Nunes-Xavier et al., 2019a). Here, we provide an update on the role of the classic family of PTPs in neuroblastoma. We have focused on those PTPs reported to regulate neuroblastoma cell growth, survival, or differentiation, or whose expression it has been reported to be affected in neuroblastoma cells upon different cell growth conditions. Insights are made on specific PTPs as potential neuroblastoma biomarkers and molecular therapeutic targets.

## Molecular Signaling Pathways in Neuroblastoma

**FIGURE 1:**
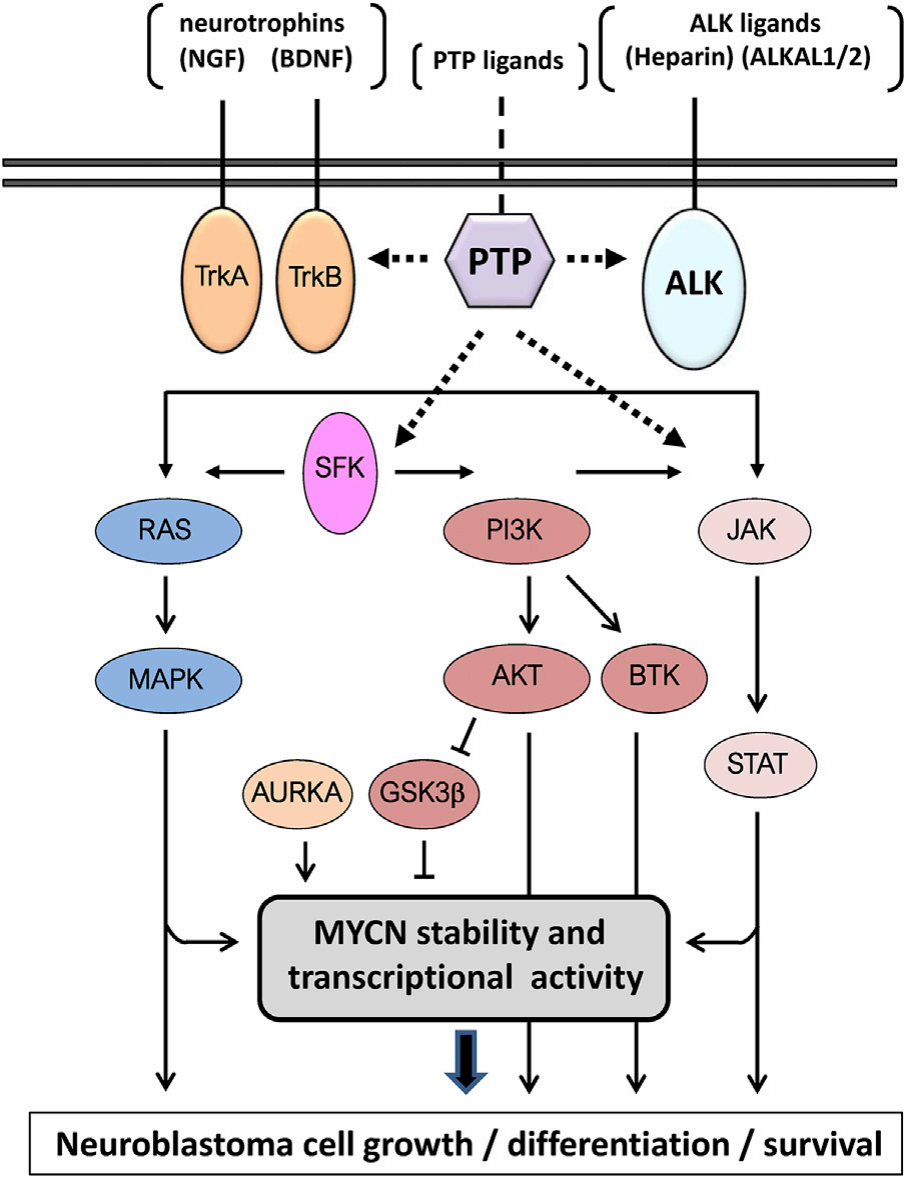
Schematic depiction of the major signaling pathways in neuroblastoma cells driven by Trk and ALK receptors upon ligand binding. The potential participation of classical protein tyrosine phosphatases (PTPs) is indicated by dashed lines. Note that the same depiction is used for both receptor-like and non-receptor PTPs. The pathways converge in the regulation of MYCN (N-Myc) functions. See text for more details.

The amplification of *MYCN* gene is the most frequent genomic alteration in neuroblastoma, in association with poor cell differentiation and bad prognosis, which occurs in about 25% of primary neuroblastoma tumors and constitutes the major hallmark of high-risk neuroblastoma (Huang and Weiss, 2013; Tolbert and Matthay, 2018; Westermark et al., 2011). *MYCN* gene encodes the MYCN (N-Myc) transcription factor, whose expression and functional activity is highly regulated in the nervous system during embryonic and early-life development, playing an important role in the maintenance of neural stem cell pluripotency and in the differentiation of neural progenitors (Hurlin, 2005; Ruiz-Pérez et al., 2017). High expression of MYCN protein associates with highly aggressive neuroblastoma in a group of patients, and overexpression of MYCN in developing neuroblasts spontaneously triggers the growth of aggressive undifferentiated neuroblastoma tumors (Kamili et al., 2020; Wang et al., 2015). Thus, MYCN activity drives a major gene expression-regulatory node which is under the control of the major neuroblastoma cell signaling pathways. These signal transduction pathways are often activated upon binding of Trk- and ALK-receptor tyrosine kinases (RTKs) to their ligands, and mainly include the RAS/MAPK, PI3K/AKT, and JAK/STAT pro-oncogenic pathways (Figure 1). Accordingly, pharmacological inhibitors of Trk and ALK RTKs are being tested in clinical trials in neuroblastoma (Lange and Lo, 2018; Pacenta and Macy, 2018; Higashi et al., 2019).

Trks constitute a family of neurotrophin receptors with multiple and complementary roles in the nervous system. Trk family members include TrkA (NTRK1), TrkB (NTRK2), and TrkC (NTRK3), whose neurotrophin binding preference is nerve growth factor (NGF), brain-derived neurotrophic factor and neurotrophin-4/5 (BDNF, NT4/5), and neurotrophin-3 (NT3), respectively (Barbacid, 1994; Brodeur et al., 2009; Cocco et al., 2018). No relevant incidence of mutations has been found in neuroblastoma for *NTRK1*, *NTRK2*, or *NTRK3* genes (https://cancer.sanger.ac.uk; https://pecan.stjude.cloud). TrkA and TrkC high expression are favorable prognostic markers in neuroblastoma, in association with a differentiated phenotype and absence of MYCN amplification, whereas high TrkB expression associates with aggressive neuroblastomas and chemotherapy resistance (Nakagawara et al., 1993; Nakagawara et al., 1994; Yamashiro et al., 1997; Ho et al., 2002; Jaboin et al., 2002). This relates with the differential roles of these RTKs in neuronal function: NGF/TrkA signaling stimulates sympathetic neuron differentiation, whereas BDNF/TrkB signaling facilitates neuronal plasticity, survival and angiogenesis (Schramm et al., 2005). In this regard, expression of TrkA and TrkC in the absence of their ligands triggers neuronal apoptosis, which has been associated with spontaneous neuroblastoma regression (Bouzas-Rodriguez et al., 2010; Nikoletopoulou et al., 2010; Brodeur, 2018), although it should be mentioned that the expression of the alternative TrkA splice variant TrkAIII associates with advanced neuroblastoma, and TrkAIII displays oncogenic properties in neuroblastoma cell models (Farina et al., 2018). A complex scenario emerges in which the temporal relative expression and regulation of the function of each Trk and neurotrophin ligand selectively drive pro-oncogenic or anti-oncogenic intracellular signaling during neuroblastoma progression.

*ALK* is the gene more frequently mutated in sporadic neuroblastoma, as well as in the germline of familial neuroblastoma patients. Most of these mutations are gain-of-function missense mutations which target the intracellular catalytic domain of ALK and generate a constitutively active RTK (Ogawa et al., 2011; Carpenter and Mossé, 2012; Janoueix-Lerosey et al., 2018; Trigg and Turner, 2018). ALK mutations have been associated with poorer survival in high-risk neuroblastoma, and *ALK* gene is amplified in a subgroup of *MYCN*-amplified neuroblastoma tumors due to the proximity of the two loci (2p23-24) at the 2p-gain region associated with high-risk neuroblastoma (De Brouwer et al., 2010; Bresler et al., 2014). This, together with the findings that MYCN and ALK participate in a positive feedback transcriptional regulatory loop (Schönherr et al., 2012; Hasan et al., 2013), sustains a cooperative pro-oncogenic effect of MYCN and ALK in human neuroblastoma. ALK activating ligands include heparin and ALKAL1/2 (FAM150A/B; AUGβ/α) (Guan et al., 2015; Murray et al., 2015; Reshetnyak et al., 2015), and *ALKAL2* gene is also located within the 2p-gain region, specifically at 2p25 (Javanmardi et al., 2019). In the absence of ALK mutations, overexpression of ALKL2 potentiates MYCN-driven neuroblastoma in mice, emphasizing the importance of ALK as a therapeutic neuroblastoma target (Borenäs et al., 2021).

As other tyrosine kinase receptors, Trk and ALK are subjected to phosphorylation at multiple tyrosine residues at their intracellular catalytic domains, mainly due to auto- and *trans*-phosphorylation mechanisms, which regulate their biological activity (Reichardt, 2006; Palmer et al., 2009; Roskoski, 2013). However, specific tyrosine dephosphorylation of Trk and ALK receptors in neuroblastoma is poorly documented. Other relevant tyrosine-phosphorylated effectors in the propagation of the oncogenic cell signaling in neuroblastoma cells, which also constitute potential therapeutic targets and PTP direct substrates, include Src-family kinases (SFK) (Bieerkehazhi et al., 2017; Molinari et al., 2018), JAK and STAT kinases (Yan et al., 2013; Yogev et al., 2019), Bruton tyrosine kinase (BTK) (Li et al., 2018; Pikatan et al., 2020), and MYCN stabilizing Aurora-A kinase (AURKA) (Brockmann et al., 2013; Roeschert et al., 2021), among others (Figure 1).

## Classical Protein Tyrosine Phosphatases in Neuroblastoma

**FIGURE 2:**
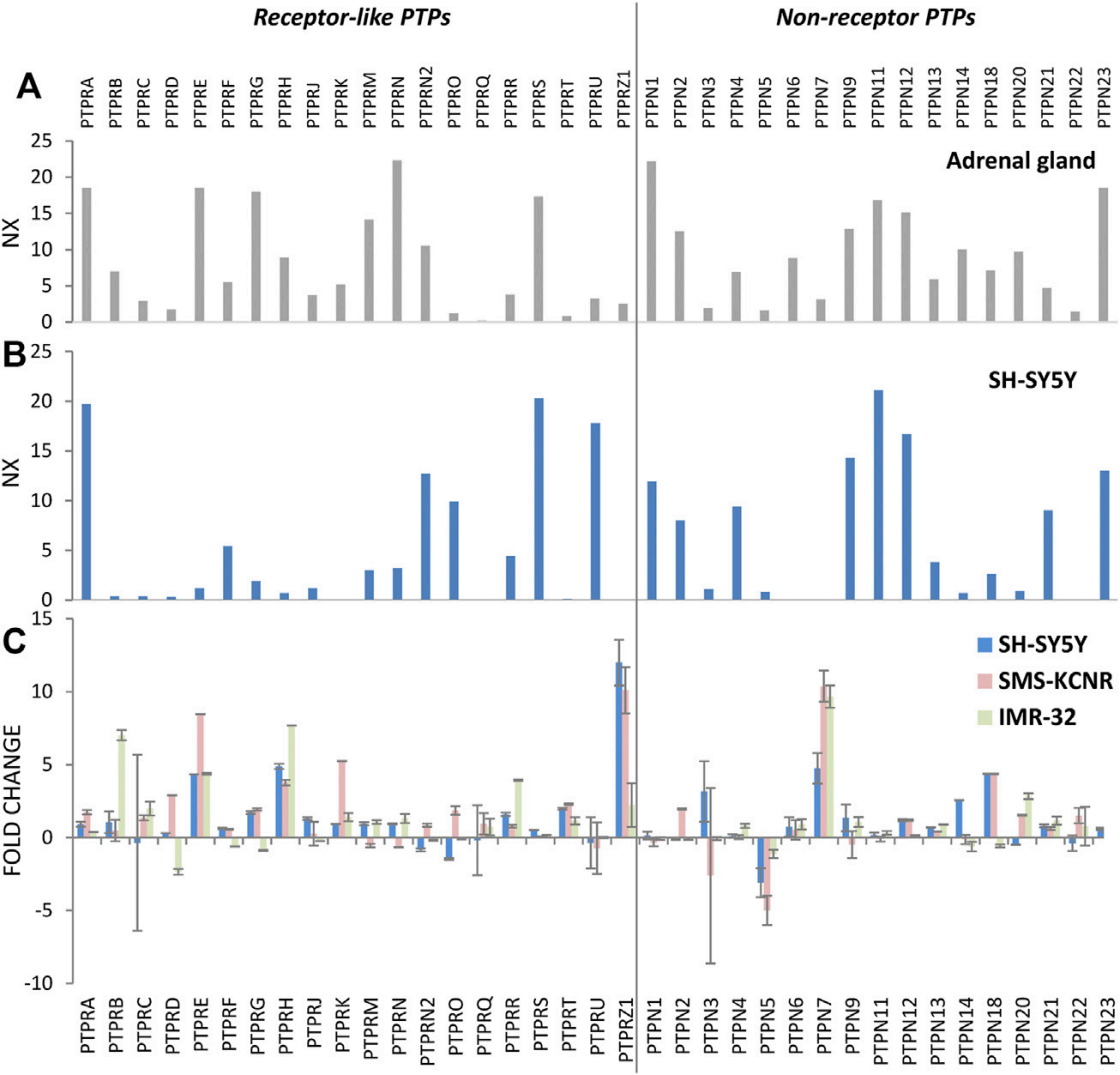
mRNA expression of classical protein tyrosine phosphatases (PTPs) in adrenal gland and in neuroblastoma cell lines. **(A)** mRNA expression in adrenal gland. RNA-seq data is shown as consensus normalized eXpression (NX) by combining HPA, GTEx and FANTOM5 datasets (https://www.proteinatlas.org/). **(B)** mRNA expression in SH-SY5Y human neuroblastoma cells. RNA-seq data is shown as consensus normalized eXpression (NX) by combining HPA, GTEx and FANTOM5 datasets (https://www.proteinatlas.org/). **(C)** mRNA expression analysis from SH-SY5Y, SMS-KCNR, and IMR-32 human neuroblastoma cells treated with retinoic acid (RA). Cell lines were kept untreated or were treated for 10 days with 10 μM RA, mRNA was purified and RT-qPCR was performed using a set of classical PTPs primers, as described in (Nunes-Xavier and Pulido, 2016). Relative mRNA expression values are shown in Log2 as fold change ±S.D. of treated cells versus untreated cells, from at least two independent experiments. Quantifications were normalized to the HPRT1 reference gene data, and mean fold change above 2 or below -2 was considered significant using Pearson Chi square analysis, as reported (Nunes-Xavier et al., 2019b).

**FIGURE 3:**
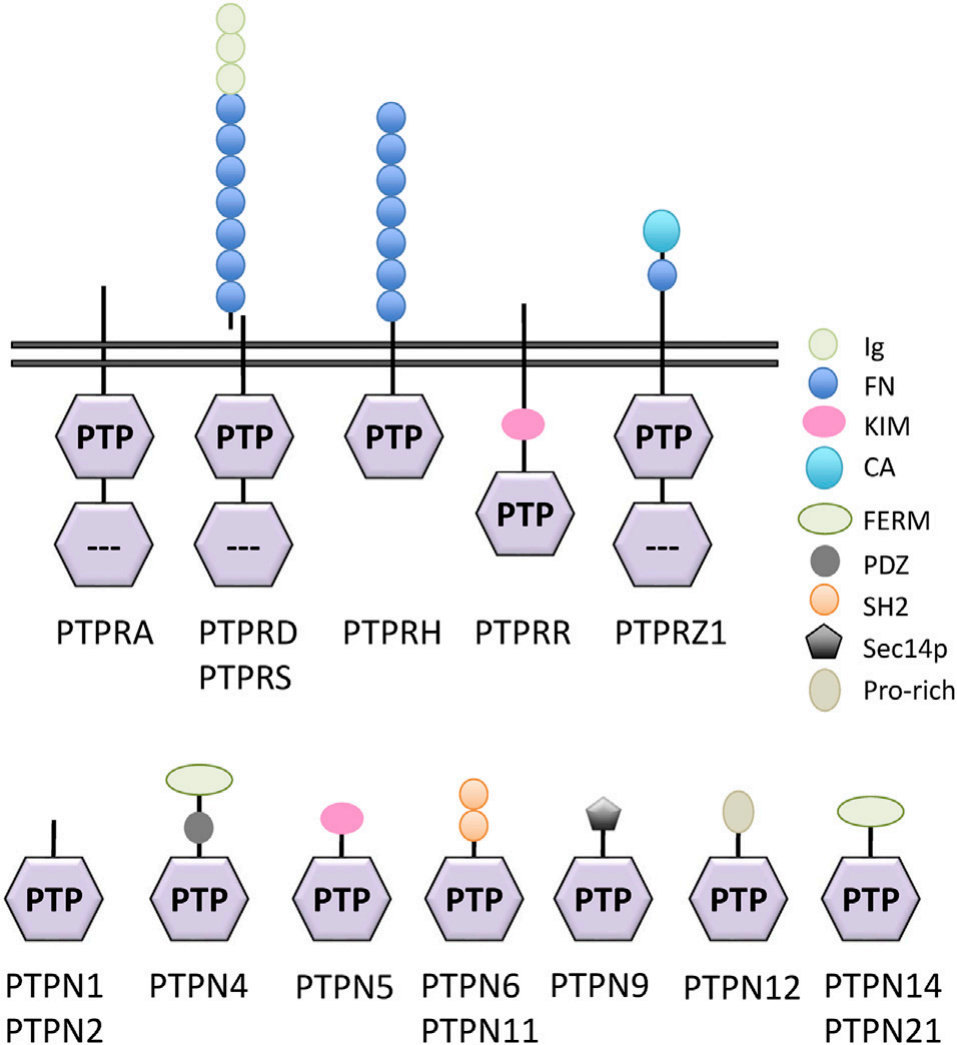
Schematic depiction of classical protein tyrosine phosphatases (PTPs) with potential involvement in neuroblastoma. In the top, receptor-like PTPs are depicted; in the bottom, non-receptor PTPs are depicted. Note that both PTPRR and PTPN5 have receptor-like and non-receptor isoforms (not depicted). The domain composition of each protein is indicated. CA, carbonic anhydrase-like; FERM, band 4.1/ezrin/radixin/moesin homology; FN, Fibronectin III-like; IG, immunoglobulin-like; KIM, kinase interaction motif; PDZ, postsynaptic density-95/discs large/ZO1 homology; Pro, proline-rich; PTP, catalytically active protein tyrosine phosphatase; Sec14, lipid-binding Sec14p homology; SH2, Src-homology 2. The C-terminal PTP domain from receptor-like PTPs containing two PTP domains either has low activity or is inactive, and it is crossed with dots.

**FIGURE 4:**
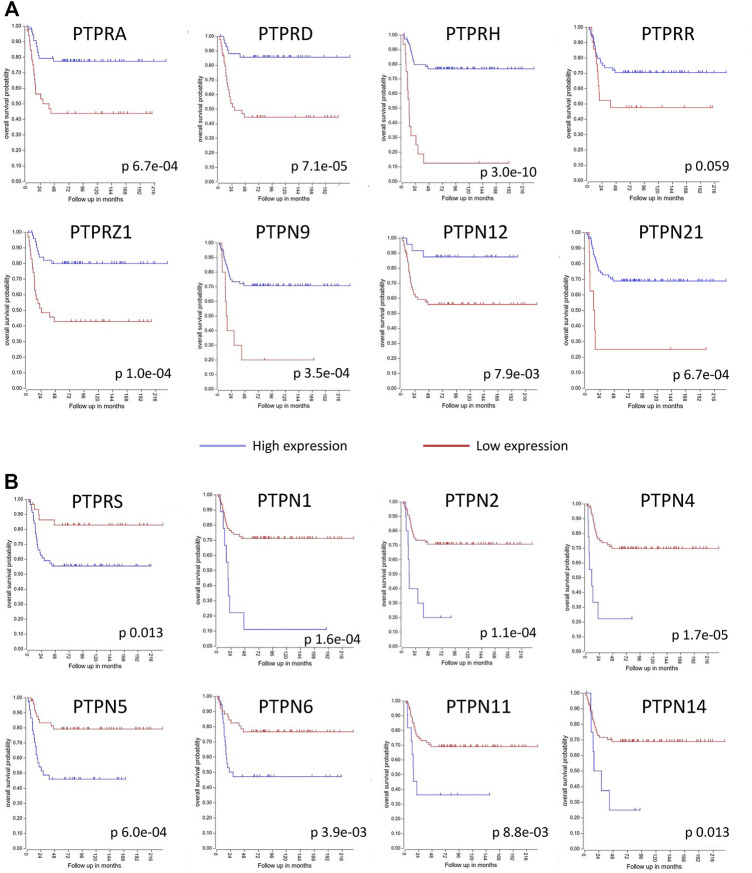
Kaplan-Meier plots of neuroblastoma patient overall survival in relation with protein tyrosine phosphatases (PTPs) mRNA expression. PTPs listed in Table 1 are included. Blue and red lines indicate high expression and low expression, respectively. In *x* axis, overall survival probability; in *y* axis, follow up in months. Data is from study Tumor Neuroblastoma public - Versteeg - 88 - MAS5.0 - u133p2 (number of patients = 88). **(A)** PTPs whose low expression associates with lower overall survival (*p* < 0.05, except for PTPRR). **(B)** PTPs whose high expression associates with lower overall survival (*p* < 0.05). Plots are from R2: Genomics Analysis and Visualization Platform (http://r2.amc.nl).

Classical PTPs are defined by the presence of a classical PTP catalytic domain, which displays the conserved HCxxGxxR signature motif containing the catalytic cysteine residue, and they can be broadly classified into two major groups: receptor-like (RPTP, PTPR) and non-receptor enzymes (NRPTP, PTPN). Receptor-like PTPs (20 genes in human genome) harbor a transmembrane and an extracellular region capable of ligand binding, and possess in most of the cases two classic PTP domains located in the cytoplasm and positioned in tandem, one with catalytic activity (D1) and one with regulatory activity (D2). Non-receptor PTPs (17 genes in human genome) are non-transmembrane proteins harboring a single classic PTP cytoplasmic domain, as well as additional regulatory domains important for function and subcellular location (Hendriks et al., 2013; Alonso and Pulido, 2016; Mohebiany et al., 2013; Tonks, 2006). The mRNA expression profiles of the two groups of human classical PTPs in the adrenal gland and in the SH-SY5Y human neuroblastoma cell line are shown in Figures 2A,B, as retrieved from public databases. Retinoic acid induces differentiation of neuroblastoma cells, and it is used as a maintenance therapy agent in high-risk neuroblastoma patients (Bayeva et al., 2021). Figure 2C shows the changes of mRNA expression of human classical PTPs from the human neuroblastoma cell lines SH-SY5Y, SMS-KCNR, and IMR-32 undergoing retinoic acid-induced differentiation. Substantial alterations in the mRNA expression levels of several classical PTPs are observed, arguing for the involvement of members of this group of enzymes in the modulation of neuroblastoma growth and differentiation. A summary of the expression, alterations, and functional effects of selected classical PTPs in neuroblastoma is provided in Table 1, and schematic depictions of classical PTPs with relevance in neuroblastoma are illustrated in Figure 3. Kaplan-Meier plots of neuroblastoma patient survival in relation with mRNA expression of PTPs from Table 1 is shown in Figure 4, as retrieved from R2: Genomics Analysis and Visualization Platform (http://r2.amc.nl). As illustrated, high mRNA expression of a group of PTPs associates with low patient survival, suggesting pro-oncogenic roles for the product of these genes. Conversely, several PTPs display low mRNA expression in association with low patient survival, suggesting tumor suppressive roles for them in neuroblastoma. Protein expression analysis of the distinct PTPs in neuroblastoma tumor samples is required for further validation of these observations.

**TABLE 1:** Protein tyrosine phosphatases in neuroblastoma cell growth, survival and differentiation.

Gene/Protein	Alterations/functional effects in NB cell lines and NB tumors
*PTPRA*/RPTPα	- P19 cells: ↑ PTPRA mRNA upon cell aggregation; P19 cells overexpressing PTPRA: ↑ neuronal differentiation, ↑ Src activity (den Hertog et al., 1993)
	- N1E-115 cells: ↑ PTPRA mRNA upon DMSO differentiation (den Hertog et al., 1993)
	- NB8 cells overexpressing catalytically inactive PTPRA: ↓ cell spreading and migration, ↓ Src activity (Wu and Song, 2018)
	-SH-SY5Y cells: ↓ PTPRA protein upon CPP overexpression (Carotenuto et al., 2013)
*PTPRD*/PTPRδ	- *PTPRD* gene deleted or aberrantly spliced in neuroblastoma cell lines and tumors (Stallings et al., 2006; Nair et al., 2008)
	- ↓ PTPRD mRNA in high stage neuroblastoma tumors *vs* low stage or normal fetal adrenal neuroblasts (Nair et al., 2008)
	- Low expression levels of PTPRD mRNA and protein in neuroblastoma cell lines and in mouse embryo adrenal glands. PTPRD overexpression in neuroblastoma cell lines: no effect on cell growth or colony formation (Clark et al., 2012)
	- Low PTPRD mRNA levels associate with poor overall patient survival. PTPRD overexpression in neuroblastoma cell lines: ↓ cell growth, ↓ AURKA stabilization, ↓ MYCN protein (Meehan et al., 2012)
*PTPRS*/PTPRσ	- High expression in neuroblastoma cell lines (Clark et al., 2012)
	- SH-SY5Y cells: ↑ neurite outgrowth upon treatment with anti-PTPRS mAb (Wu et al., 2017)
	- PC12 cells: ↑ neurite outgrowth after chondroitin sulfate proteoglycan incubation upon treatment with PTPRS pharmacological inhibitors (Lee et al., 2016)
	- PC12 cells overexpressing PTPRS: ↓ NGF-induced neurite outgrowth (Chagnon et al., 2010)
*PTPRH*/SAP-1	- Neuroblastoma cell lines: ↑ PTPRH mRNA upon RA differentiation. High expression of PTPRH protein in neuroblastoma tumors, in association with poor prognosis (Nunes-Xavier et al., 2019b)
*PTPRR*/PCPTP1	- PC12 cells overexpressing cytosolic PTPRR isoform: ↓ EGF-induced pERK1/2 (Noordman et al., 2006)
	- Neuro-2a cells: shift of PTPRR from membrane- to cytoskeletal and nuclear fractions upon serum depletion differentiation (van Ham et al., 2005)
*PTPRZ1*/RPTPβ/ζ	- PC12 cells overexpressing PTPRZ1: ↓ NGF-induced neurite outgrowth (Shintani and Noda, 2008)
	- B103 cells: ↑ p190 RhoGAP tyrosine phosphorylation upon PTN stimulation (Tamura et al., 2006)
	- B103 cells: ↑ GIT1 tyrosine phosphorylation upon PTN stimulation (Kawachi et al., 2001)
	- Neuro-2a cells overexpressing DNER: ↑ DNER tyrosine phosphorylation, and ↑ RA-induced neurite outgrowth upon PTN treatment (Fukazawa et al., 2008)
	- SH-SY5Y cells: ↑ TrkA and ALK tyrosine phosphorylation and ↓ cell viability upon PTPRZ1 pharmacological inhibition. ↓ citotoxicity induced by MPP+ upon PTN stimulation (Fernández-Calle et al., 2019; Del Campo et al., 2021)
	- SH-SY5Y cells overexpressing glycosyltransferase GnT-Vb: ↑ cell surface expression of PTPRZ1 inactive protein, ↑ β-catenin tyrosine phosphorylation (Abbott et al., 2008)
	- High expression of PTPRZ1 protein in neuroblastoma tumors. In neuroblastoma cell lines: ↑ PTPRZ1 mRNA upon RA differentiation (Nunes-Xavier et al., 2019b)
*PTPN1*/PTP1B	- SH-SY5Y cells: ↑ PTPN1 mRNA upon insulin or leptin treatment; SH-SY5Y cells overexpressing PTPN1: ↓ insulin- or leptin-induced pJAK2, pSTAT3, and pERK1/2 (Benomar et al., 2009)
	- SH-SY5Y cells treated with PTPN1 inhibitor: ↑ BDNF-induced pTrkB, pAKT, and pERK1/2. SH-SY5Y cells overexpressing PTPN1: ↓ BDNF-induced pTrkB, pAKT, and pERK1/2 (Ozek et al., 2014)
	- SH-SY5Y cells treated with PTPN1 inhibitor: ↓ pPERK and peIF2α induced by endoplasmic reticulum stress agents, ↓ cytotoxicity induced by endoplasmic reticulum stresses (Jeon et al., 2017)
	- SH-SY5Y cells upon siRNA knock-down of PTPN1: ↑ EGF-induced protein tyrosine phosphorylation, ↑ cell proliferation. High expression of PTPN1 protein in neuroblastoma tumors associates with metastasis and poor prognosis (Nunes-Xavier et al., 2019b)
*PTPN2*/TC-PTP	- IMR-32 cells overexpressing PTPN2: ↓ pervanadate-, c-Src-, forskolin-induced pC3G, ↓ forskolin-induced neurite growth (Mitra et al., 2011)
*PTPN4*/PTP-MEG1	- SH-SY5Y cells: PDZ-binding dependent PTPN4 pro-survival functions (Préhaud et al., 2010)
*PTPN5*/STEP	- SH-SY5Y cells: ↓ pSTEP (↑ STEP activity) upon treatment with adenosine A2A receptor agonist, reverted by PP2A inhibition; ↑ pSTEP upon treatment with forskolin (Mallozzi et al., 2020)
*PTPN6*/SHP1	- P19 cells: ↓ PTPN6 mRNA, ↑ PTPN6 tyrosine phosphorylation (transient) following reversion of differentiation by cell aggregation and RA. P19 cells overexpressing PTPN6: ↓ neurite outgrowth, ↑ proliferation in the presence of RA (Mizuno et al., 1997)
	- N1E-115 cells: ↑ PTPN6 phosphatase activity upon treatment with angiotensin II (Bedecs et al., 1997)
	- SH-SY5Y cells overexpressing TrkAIII and treated with PTPN6 pharmacological inhibitor: ↓ Src tyrosine phosphorylation (Tyr 527), ↑ TrkAIII tyrosine phosphorylation, ↑ apoptosis upon apoptotic TRAIL stimulation (Gneo et al., 2016)
	- Low expression of PTPN6 protein in neuroblastoma tumors, in association with high TrkA tyrosine phosphorylation, associates with relapse-free survival (Youssef et al., 2019)
*PTPN9*/PTP-MEG2	- P19 cells: ↓ pTrkA (Tyr490/674/675), ↓ neurite outgrowth (Zhang et al., 2016)
*PTPN11*/SHP2	- *PTPN11* gene mutated with relatively high frequency in high-risk neuroblastoma (Bentires-Alj et al., 2004; Pugh et al., 2013; Gröbner et al., 2018; Ma et al., 2018)
	- SH-SY5Y cells overexpressing PTPN11: ↓ pTrkB (Tyr515), ↑ ER stress response, ↑ apoptosis, ↓ proliferation, ↓ neurite outgrowth. SH-SY5Y cells upon shRNA-knock-down of PTPN11: ↑ pTrkB (Tyr515), ↑ neurite outgrowth (Chitranshi et al., 2017)
	- NBFL cells overexpressing PTPN11 DN (lacking PTP domain) and treated with CNTF: ↑ CNTF-mediated gene expression response, ↑ STAT/DNA complexes, ↓ AP-1 binding activity, ↓ c-fos expression (Servidei et al., 1998)
	- High expression of PTPN11 mRNA in high-risk neuroblastoma, in association with high GAB1 mRNA expression, associates with poorer survival of patients with *MYCN* amplification (Zhang et al., 2017)
	- Neuroblastoma cell lines upon PTPN11 pharmacological inhibition: ↓ pERK1/2, ↓ proliferation (Valencia-Sama et al., 2020)
	- Neuroblastoma cell lines: physical interaction and phosphorylation of PTPN11 (Tyr542) by ALK. ↓ pERK1/2, ↓ cell growth upon pharmacological inhibition of PTPN11 (Uçkun et al., 2021)
	- SH-SY5Y cells upon Cbl/Cbl-b ubiquitin ligases siRNA knock-down: ↑ PTPN11 protein expression, ↑ pERK1/2 and neurite outgrowth (diminished by PTPN11 pharmacological inhibition) (Pedersen et al., 2021)
*PTPN12*/PTP-PEST	- SH-SY5Y cells upon PTPN12 siRNA knock-down: ↑ p130Cas, FAK, TrkB (Tyr816) tyrosine phosphorylation, ↑ pERK1/2 and neurite outgrowth (Ambjørn et al., 2013)
*PTPN14*/PTPD2	- *PTPN14* gene mutated in relapsed neuroblastoma (Schramm et al., 2015)
	- SK-N-SH cells upon ectopic expression of PTPN14 mutant derived from neuroblastoma: ↑ YAP nuclear accumulation and colony formation. (Schramm et al., 2015)
	- SK-N-BE (2) cells upon stathmin shRNA knock-down: ↓ PTPN14 mRNA and protein expression. SK-N-BE (2) and SH-SY5Y upon PTPN14 siRNA knock-down: ↑ cell migration and invasion (Po’uha et al., 2020)
*PTPN21*/PTPD1	- PC12 cells: ↓ proliferation, ↑ apoptosis (triggered by oxygen/glucose deprivation) upon siRNA PTPN21 knock-down. ↑ proliferation, ↓ apoptosis and ↑ pERK1/2 (triggered by oxygen/glucose deprivation) upon PTPN21 overexpression (Cui et al., 2017)

Cell lines: B103, rat neuroblastoma; IMR-32, human neuroblastoma; N1E-115, mouse neuroblastoma; NB8, human neuroblastoma; NBFL, human neuroblastoma; Neuro-2a, mouse neuroblastoma; P19, mouse embryonic teratocarcinoma; PC12, rat pheochromocytoma; SH-SY5Y, human neuroblastoma; SK-N-BE (2), human neuroblastoma. ALK: anaplastic lymphoma kinase, CNTF: ciliary neurotrophic factor, CPP: competitive permeable peptide impairing Nm23-H1/h-Prune interaction, C3G, guanine nucleotide exchange factor C3G, DN: dominant negative, EGF: epidermal growth factor, ER: endoplasmic reticulum, mAb: monoclonal antibody, NB: neuroblastoma, NGF: nerve growth factor, PTN: pleiotrophin, RA: retinoic acid. ↑, increase, ↓, decrease.

### Receptor-Like Protein Tyrosine Phosphatases in Neuroblastoma

**PTPRA** (RPTPα) has been documented to be involved in neuronal differentiation, as well as in neuroblastoma cell motility, in association with dephosphorylation and activation of Src (den Hertog et al., 1993; Wu and Song, 2018), a PTPRA substrate shared by other cell types (Pallen, 2003). PTPRA is highly expressed in SH-SY5Y neuroblastoma cells, whereas the related PTPRE (RPTPε) is expressed at low levels but induced upon retinoic acid differentiation (Figure 2). The possibility exists that the use of PTPRA/PTPRE inhibitors could complement the use of Src inhibitors in neuroblastoma therapies (Navarra et al., 2010; Kratimenos et al., 2014; Tintori et al., 2015). Although *PTPRA* gene mutations in neuroblastoma are uncommon, a missense variant in the PTPRA PTP D1 domain has been associated with low survival of patients (Esposito et al., 2018).

PTPRD (RPTPδ) and PTPRS (RPTPσ), together with PTPRF (LAR) belong to a subfamily of classical receptor-like PTPs which display large extracellular regions susceptible to proteolytic shedding and containing tandem repeats of fibronectin type III- and immunoglobulin-like domains (Figure 3) (Chagnon et al., 2004; Pulido et al., 1995). A major physiologic role for these PTPs involves the regulation of synapsis formation and function (Han et al., 2016; Um and Ko, 2013). In the context of cancer disease, **PTPRD** is absent or inactive in a variety of human tumor types, playing a tumor suppressor role linked with the downregulation of STAT3 activity (Lin et al., 2021; Ortiz et al., 2014; Solomon et al., 2008; Veeriah et al., 2009). A tumor suppressor role has also been proposed for PTPRD in neuroblastoma, with a relatively high incidence of gene losses and gene fusions targeting the *PTPRD* gene in neuroblastoma samples (https://pecan.stjude.cloud) (Stallings et al., 2006; Nair et al., 2008). Low expression of PTPRD mRNA associates with poor disease progression and neuroblastoma patient survival, and a direct role has been proposed for PTPRD in AURKA dephosphorylation and destabilization in neuroblastoma cells (Nair et al., 2008; Meehan et al., 2012). However, no evidence of PTPRD tumor suppressor activity in neuroblastoma cells has also been reported (Clark et al., 2012). It should be interesting to test the potential regulatory role of PTPRD in the JAK/STAT pathway in neuroblastoma cells.

Distinctly to PTPRD, the related **PTPRS** enzyme is highly expressed in neuroblastoma cells (Clark et al., 2012) (Figure 2). A role for PTPRS in tyrosine dephosphorylation of Trk receptors has been shown in primary sensory neurons (Faux et al., 2007), and synapse signaling-inducing binding between the extracellular regions of PTPRS and TrkC has been reported (Takahashi et al., 2011). A mAb against the ectodomain of PTPRS promotes neurite outgrowth in SH-SY5Y cells (Wu et al., 2017), and PTPRS pharmacological inhibitors revert the inhibitory neurite outgrowth effect of chondroitin sulfate proteoglycan in PC12 cells (Lee et al., 2016). Conversely, overexpression of PTPRS inhibited NGF-induced PC12 axonal outgrowth (Chagnon et al., 2010). These findings support a negative role for PTPRS in neurite extension/neuroblastoma cell differentiation. **PTPRF** has also been functionally associated with TrkB and neurotrophic signaling in embryonic neurons (Yang et al., 2006). *PTPRF* gene maps at 1p34, within a region frequently deleted in high-risk neuroblastoma (Bown, 2001). Whether PTPRS or PTPRF may directly regulate the tyrosine phosphorylation of Trk receptors or other specific substrates in neuroblastoma cells, deserves experimental analysis.

**PTPRH** (SAP-1) belongs to a subfamily of receptor like-PTPs with a large extracellular region enriched in fibronectin type III-like domains and a single intracellular catalytic classical PTP domain (Figure 3). Other members of this subfamily include PTPRB, PTPRJ, PTPRO, and PTPRQ (Chicote et al., 2017; Jeon and Zinn, 2015; Matozaki et al., 2010). PTPRH protein is highly expressed in neuroblastomas, in association with tumor low stage and patient low risk, and PTPRH mRNA is induced in neuroblastoma cells upon retinoic acid differentiation (Nunes-Xavier et al., 2019b) (Figure 2). These findings suggest a role for PTPRH in differentiation programs limiting neuroblastoma cell growth, and are consistent with the upregulation of PTPRH expression upon differentiation of other cell types (Nunes-Xavier et al., 2012; Nunes-Xavier et al., 2013), as well as with its downregulation in some cancer types (Nagano et al., 2003; Bujko et al., 2017). Remarkably, PTPRH was selected in a siRNA screening as a potential STAT3 regulator (Parri et al., 2020), and it has also been found to associate with and dephosphorylate several RTK, including EGFR and IR (Shintani et al., 2015; Yao et al., 2017). Since the related PTPRO enzyme, which is mainly expressed in the developing nervous system, dephosphorylates Trk receptors (Hower et al., 2009; Gatto et al., 2013), a role is possible for PTPRH in the direct regulation of Trk signaling in neuroblastoma cells. **PTPRQ** is a unique classical PTP because it specifically dephosphorylates phosphatidylinositide substrates (Oganesian et al., 2003). It is of interest that the *PTPRQ* gene is among the few PTP genes that have been found mutated with relative high frequency in neuroblastoma tumor samples (https://pecan.stjude.cloud), although the functional consequences of these mutations need to be evaluated.

PTPRR (PCPTP1, PTP-SL), PTPN5 (STEP), and PTPN7 (HePTP, LC-PTP), belong to the subgroup of kinase interaction motif (KIM)-containing classical PTPs, whose major substrates are the MAPK ERK1/2 and p38s (Barr and Knapp, 2006; Pulido et al., 1998; Torres et al., 2004). A major negative regulatory mechanism of these PTPs is PKA-mediated phosphorylation of their KIM, which abrogates substrate binding (Blanco-Aparicio et al., 1999). The *PTPRR* and *PTPN5* genes are mainly expressed in the brain and encode transmembrane and non-transmembrane PTP isoforms, although PTPN5 was originally classified as a non-transmembrane PTP (Hendriks et al., 2009; Karasawa and Lombroso, 2014). PTPN7 is a non-transmembrane PTP expressed predominantly in hemopoietic cells (Zanke et al., 1992). **PTPRR** mRNA (but not PTPN5 or PTPN7 mRNAs) was expressed in PC12 cells, and cytosolic PTPRR expression down-regulated ERK1/2 phosphorylation (Noordman et al., 2006). In SH-SY5Y cells, **PTPN5** KIM phosphorylation was shown to be regulated through adenosine A2A receptor and the countereffects of PKA and PP2A (Mallozzi et al., 2020). Interestingly, a distinct pattern of PTPRR, PTPN5, and PTPN7 mRNA expression was observed in human neuroblastoma cell lines upon retinoic acid differentiation, with down-regulation of PTPN5 and up-regulation of PTPN7 and, to a lesser extent, PTPRR (Figure 2). These observations support the notion that MAPK activation status is finely tuned during neuroblastoma cells differentiation by the action of specific PTPs, which could impact on tumor development. In line with this, the expression of the dual-specificity MAPK phosphatase DUSP5 associates with neuroblastoma relapse and poor prognosis (Aurtenetxe et al., 2018). Since activating mutations in components of the RAS/MAPK pathway are frequent in relapsed neuroblastoma (Huang et al., 2013), therapeutic targeting of this pathway is currently being tested in neuroblastoma patients (Mlakar et al., 2021). Whether the KIM-containing classical PTPs may play a physiologic role in the control of MAPK activity in neuroblastomas deserves dedicated analysis.

**PTPRZ1** (RPTPζ, RPTPβ/ζ) is a receptor-like PTP predominantly expressed in the central nervous system and in endothelial tumor cells. Its extracellular domains include a carbonic anhydrase-like domain, a fibronectin type III-like domain, and a carbohydrate-rich region (Figure 3). Alternative splicing generates a shorter transmembrane protein lacking the glycosylated region, as well as two soluble extracellular isoforms, the chondroitin sulfate proteoglycans phosphacans (Pantazaka and Papadimitriou, 2014; Papadimitriou et al., 2016). A wide array of ligands and substrates have been described for PTPRZ1 in a variety of cell types, which could account for the many cell-cell adhesion and communication, cell migration, and cell growth functions attributed to this PTP. Major PTPRZ1 ligands include the heparin-binding growth factors pleiotrophin (PTN) and midkine (MK), the extracellular matrix proteins tenascins (TN), and cell adhesion contactin (CNTN) family members, among others. Several PDZ domain-containing proteins, such as MAGIs and DLGs, associate intracellularly with PTPRZ1 through its PDZ-binding motif. PTPRZ1 substrates with potential relevance in neuroblastoma include ALK, TrkA, p190 RhoGAP, β-catenin, and SFK, among others (Herradon and Ezquerra, 2009; Mohebiany et al., 2013; Xia et al., 2019). PTN binding to PTPRZ1 inhibits its catalytic activity by triggering PTPRZ1 oligomerization. In neuroblastoma cells, this leads to increased tyrosine phosphorylation of p190 RhoGAP, GIT1, and DNER (Kawachi et al., 2001; Tamura et al., 2006; Fukazawa et al., 2008), and it has been proposed that the PTN inhibitory effect on PTPRZ1 acts as a major ALK indirect activating mechanism (Deuel, 2013). In addition, regulated glycosylation of PTPRZ1 in neuroblastoma cells also induces its dimerization and catalytic inactivation (Abbott et al., 2008), suggesting a tight physiologic control of constitutive PTPRZ1 catalytic activity. In this regard, PTPRZ1 protein is abundantly expressed in neuroblastoma tumors, and PTPRZ1 mRNA expression is induced in neuroblastoma cell lines upon cell differentiation, although its expression in adrenal gland or in neuroblastoma cells under normal cell growth conditions seems to be very low, making difficult a quantitative comparison (Nunes-Xavier et al., 2019b). PTN mRNA expression in neuroblastoma correlates with favorable prognosis (Nakagawara et al., 1995), and in an *in vivo* neuroblastoma xenograft model, *PTN* gene expression was found down-regulated in tumors resistant to irinotecan therapy, as compared to sensitive tumors (Calvet et al., 2006). Small-molecule inhibitors of PTPRZ1 have been proposed as suitable therapeutic alternatives for glioblastoma and central nervous system disorders (Fujikawa et al., 2016; Fujikawa et al., 2017; Pastor et al., 2018), and pharmacological inhibition of PTPRZ1 resulted in increased ALK and TrkA phosphorylation in SH-SY5Y neuroblastoma cells (Fernández-Calle et al., 2018). Modulation of PTPRZ1 activity using specific ligands or pharmacological inhibitors could be an alternative treatment for neuroblastoma that needs to be explored.

### Non-Receptor Protein Tyrosine Phosphatases in Neuroblastoma

**PTPN1** (PTP1B), the founding member of the PTP gene family (Tonks, 2013), is an ubiquitously expressed negative regulator of insulin signaling, and it plays both oncogenic and tumor suppressor roles in human cancer (Bakke and Haj, 2015; Feldhammer et al., 2013). This makes PTPN1 a potential therapeutic target for human disease, including human malignancies (Kostrzewa et al., 2019; Sharma et al., 2020). A hydrophobic C-terminal sequence targets PTPN1 to the cytoplasmic face of the endoplasmic reticulum, which determines PTPN1 access to protein substrates during biosynthetic, endocytic, or cell signaling pathways. Potential PTPN1 substrates relevant in neuroblastoma include, among others, RTK, JAK/STAT, SFK, p130Cas, and PERK. PTPN1 has been linked to tyrosine dephosphorylation of the intracellular pool of ALK in mouse fibroblasts (Boutterin et al., 2013), raising the possibility that PTPN1 may dephosphorylate ALK in neuroblastoma cells. Knock-down of PTPN1 in SH-SY5Y cells increased the protein phospho-tyrosine content upon EGF stimulation, as well as cell proliferation, whereas high PTPN1 protein expression in neuroblastoma tumors associated with poor prognosis (Nunes-Xavier et al., 2019b). This suggests a regulatory role for PTPN1 in transformation of neuroblastoma cells. Using different *in vitro* SH-SY5Y cellular models, a role for PTPN1 in the dephosphorylation of JAK2, STAT3, ERK1/2, TrkB, and AKT has been proposed (Benomar et al., 2009; Jeon et al., 2017). It will be important to define the direct substrates of PTPN1 in neuroblastoma cells upon different conditions of growth and differentiation. **PTPN2** (TC-PTP) is highly related to PTPN1. It also displays a wide tissue distribution and exists in humans as two isoforms, one nuclear and one associated to the endoplasmic reticulum (Stuible et al., 2008; Tiganis, 2013). PTPN2 has been involved in different human cancers, with a wide implication in lymphoid malignancies. This, together with its negative role in anti-tumor immunity, make PTPN2 a suitable target in oncology (Pike et al., 2016; Wiede et al., 2020). PTPN2 has been found to dephosphorylate the Rap1guanine nucleotide exchange factor C3G in SH-SY5Y cells, and to inhibit the neurite outgrowth triggered by the adenylate cyclase activator forskolin (Mitra et al., 2011). C3G is a positive regulator of differentiation and survival of neuroblastoma cells, through signaling pathways involving ALK (Radha et al., 2008; Schönherr et al., 2010). Additional PTPN2 substrates related with neuroblastoma cell growth are expected, although the expression of PTPN2 in neuroblastoma cells is poorly documented. In this regard, both PTPN1 and PTPN2 mRNA expression is detected in SH-SY5Y cells (Figure 2). In addition, PTPN1 and PTPN2 have been shown to dephosphorylate ALK and PTPN11 in anaplastic large cell lymphomas, and gene deletion of either PTPN1 or PTPN2 induced resistance to ALK inhibitors (Karaca Atabay et al., 2021). The possibility exists that ALK and PTPN11 are direct substrates of PTPN1 and PTPN2 in neuroblastoma cells.

PTPN6 (SHP1) and PTPN11 (SHP2) are structurally related PTPs which harbour two tandem regulatory Src homology 2 (SH2) phospho-tyrosine binding domains preceding the catalytic PTP domain (Figure 3). Intramolecular interaction of the N-terminal SH2 (N-SH2) domain with the PTP domain occludes the enzyme active site and keeps the protein catalytically inactive. Upon SH2-mediated binding to defined phospho-tyrosine residues in receptor or adaptor proteins, PTPN6 and PTPN11 are activated to dephosphorylate specific protein substrates. This makes these two PTPs major regulators of intracellular signaling in response to growth and differentiation factors. PTPN6 is mainly expressed in hematopoietic cells and, to a lesser extent, in endothelial cells, whereas the tissue expression of PTPN11 is ubiquitous (Chong and Maiese, 2007; Lorenz, 2009; Dempke et al., 2018). **PTPN6** overexpression in P19 cells has been linked to a decrease in neuronal differentiation and an increase in proliferation, whereas PTPN6 inhibition has been found to facilitate apoptosis of *splice variant* TrkAIII-expressing SH-SY5Y cells in a Src-mediated process (Mizuno et al., 1997; Gneo et al., 2016). This is in accordance with the association found between good patient prognosis and PTPN6 low expression and tyrosine phosphorylated TrkA (Tyr674/675) expression in neuroblastoma tumors (Youssef et al., 2019). In this regard, PTPN6 has been proposed to negatively regulate TrkA in neurons and in breast cancer cells by dephosphorylation of TrkA Tyr674/675 residues (Marsh et al., 2003; Montano, 2009).

**PTPN11** acts as an oncogenic phosphatase in several cancer types by dephosphorylation of a wide variety of effector and regulatory signaling proteins, mainly acting downstream of RTKs to promote activation of the RAS/MAPK pathway. PTPN11 direct substrates include RTKs, RAS proteins and RAS negative regulators, as well as SFKs and SFK regulators, among others (Buday and Vas, 2020; Chan et al., 2008; Grossmann et al., 2010; Matozaki et al., 2009). Accordingly, pharmacological inhibitors of PTPN11 catalysis have emerged as potential anti-cancer drugs (Shen et al., 2020; Song et al., 2021; Yuan et al., 2020), making the elucidation of the cancer type-specific PTPN11 substrates an important demand. In addition, phosphatase-independent functions, in some cases mediated by protein-protein interactions, also contribute to PTPN11 regulation of oncogenic signaling (Guo and Xu, 2020). Thus, both the catalytic activity and the expression levels of PTPN11 are relevant in human cancer. Although a wide diversity in the functional output of *PTPN11* gene disease-associated mutations exists, gain-of-function *PTPN11* mutations are relatively frequent in human cancer, mainly targeting two hot-spots encoding specific regions at the N-SH2 and PTP domains of the enzyme (https://cancer.sanger.ac.uk). In addition, *PTPN11* is mutated in the germline of patients with several developmental disorders (Noonan Syndrome [NS], and NS-related syndromes) and hematological malignancies (Juvenile Myelomonocytic Leukemia [JMML], and other childhood leukemias) (Huang et al., 2014; Tajan et al., 2015). *PTPN11* is among the genes more frequently mutated in neuroblastoma, especially in relapsed tumors, with a mutation distribution pattern similar to the one found in other human cancers (Gröbner et al., 2018; Ma et al., 2018; Pugh et al., 2013; Eleveld et al., 2015) (https://cancer.sanger.ac.uk; https://pecan.stjude.cloud). In addition, high expression of *PTPN11* mRNA associates with poorer survival of high-risk neuroblastoma patients with *MYCN* amplification (Zhang et al., 2017). This supports the notion that high expression and gain-of-function mutations at *PTPN11* enable therapy resistance and recurrence in neuroblastoma. In this regard, pharmacological inhibition of PTPN11 in neuroblastoma cells causes RAS/MAPK pathway- and cell growth inhibition, in a manner dependent on the *RAS* mutational status and synergistic with RAF/MEK/ERK inhibitors (Valencia-Sama et al., 2020; Pedersen et al., 2021; Uçkun et al., 2021). This highlights the therapeutic potential in high-risk neuroblastoma of combination therapies targeting different effectors in the RAS/MAPK pathway. PTPN11 has been functionally related with TrkB and ALK in neuroblastoma (Chitranshi et al., 2017; Uçkun et al., 2021), making possible that these RTKs are PTPN11 neuroblastoma substrates.

**PTPN14** has been shown to be involved in migration, invasion, and proliferation of neuroblastoma cells, as well as in the regulation of the nuclear translocation of the transcription coactivator YAP (Schramm et al., 2015; Po’uha et al., 2020). Mutations at *PTPN14* gene have been found specifically in relapsed neuroblastoma tumors, suggesting the existence of an operative functional axis PTPN14-YAP in neuroblastoma relapse (Schramm et al., 2015). YAP interacts with the PPxY motifs from PTPN14, which causes YAP cytoplasmic sequestration independently of PTPN14 catalytic activity. In addition, it has also been reported that YAP is a substrate of PTPN14 (Huang et al., 2013; Liu et al., 2013; Michaloglou et al., 2013). **PTPN21** is structurally related to PTPN14, and it has been shown to be involved in negative regulation of apoptosis in PC12 cells in association with ERK1/2 activation (Cui et al., 2017). The identification of PTPN14 and PTPN21 direct substrates is necessary to fully understand the role of these non-receptor PTPs in neuroblastoma.

Other non-receptor PTPs related with neuroblastoma include PTPN4, PTPN9, and PTPN12. **PTPN4** possesses a FERM and a PDZ protein domain specialized in protein-protein interactions, and the binding of PTPN4 PDZ domain with protein partners has been postulated to positively regulate cell survival in cancer cells, including neuroblastoma cells (Préhaud et al., 2010). **PTPN9** harbors a lipid-binding Sec14 domain that target the enzyme to secretory vesicles, regulating vesicle size and fusion, and PTPN9 has been shown to directly dephosphorylate TrkA in P19 neuroblastoma cells, which would regulate its transport to the plasma membrane (Zhang et al., 2016). Finally, **PTPN12** is highly expressed in SH-SY5Y cells (Figure 2), where it has been proposed to regulate cell differentiation by dephosphorylation of p130Cas, FAK, and TrkB (Ambjørn et al., 2013).

## Concluding Remarks

The involvement of PTPs in the regulation of neuroblastoma cell signaling and development mediated by the distinct RTK/MYCN axes argues for PTPs as relevant biomarkers and potential therapeutic targets in this type of cancer. This is reinforced by the differential association found between neuroblastoma patient outcome and expression of specific PTP genes in neuroblastoma tumor samples, as illustrated along this review. A prominent example of pro-oncogenic PTP in neuroblastoma is PTPN11, whose gene is mutated with relative frequency in high-risk neuroblastoma tumors and whose inhibition by both allosteric and catalytic inhibitors is under intense scrutiny in neuroblastoma experimental models. PTPs are difficult to target specifically using small molecule inhibitors. In this regard, the diversity of regulatory and protein interaction domains present in PTPs offers a wide variety of potential intervention points in addition to direct modulation of catalysis by compounds targeting the enzyme active site (Stanford and Bottini, 2017; Turdo et al., 2021). Since dual pro- and anti-oncogenic properties are proposed for several PTPs in neuroblastoma, dedicated studies are necessary that address the expression, subcellular location, and substrate specificity of individual PTPs in different neuroblastoma scenarios. The deep understanding of PTP biology during neuroblastoma cell growth and differentiation will facilitate the testing of modulation of PTP catalysis, as well as the interference with catalytically-independent PTP biological functions, as helpful strategies in the setting of novel neuroblastoma targeted therapies.
